# Reconstitution of the Ataxia-Telangiectasia Cellular Phenotype With Lentiviral Vectors

**DOI:** 10.3389/fimmu.2018.02703

**Published:** 2018-11-20

**Authors:** Diana Carranza, Sara Torres-Rusillo, Gloria Ceballos-Pérez, Eva Blanco-Jimenez, Martin Muñoz-López, José L. García-Pérez, Ignacio J. Molina

**Affiliations:** ^1^Institute of Biopathology and Regenerative Medicine, Center for Biomedical Research, University of Granada, Granada, Spain; ^2^Genomic Medicine Department, Centro Pfizer-Universidad de Granada-Junta de Andalucía de Genómica e Investigación Oncológica (GENYO), Granada, Spain; ^3^Medical Research Council Human Genetics Unit, Institute of Genetics and Molecular Medicine, Western General Hospital, University of Edinburgh, Edinburgh, United Kingdom; ^4^Instituto de Investigación Biosanitaria ibs.GRANADA, Granada University Hospitals, University of Granada, Granada, Spain

**Keywords:** Ataxia-Telangiectasia, gene therapy, lentiviral vectors, monogenic diseases, cell reconstitution, Primary immunodeficiency

## Abstract

Ataxia-telangiectasia (A-T) is a complex disease arising from mutations in the *ATM* gene (Ataxia-Telangiectasia Mutated), which plays crucial roles in repairing double-strand DNA breaks (DSBs). Heterogeneous immunodeficiency, extreme radiosensitivity, frequent appearance of tumors and neurological degeneration are hallmarks of the disease, which carries high morbidity and mortality because only palliative treatments are currently available. Gene therapy was effective in animal models of the disease, but the large size of the *ATM* cDNA required the use of HSV-1 or HSV/AAV hybrid amplicon vectors, whose characteristics make them unlikely tools for treating A-T patients. Due to recent advances in vector packaging, production and biosafety, we developed a lentiviral vector containing the *ATM* cDNA and tested whether or not it could rescue cellular defects of A-T human mutant fibroblasts. Although the cargo capacity of lentiviral vectors is an inherent limitation in their use, and despite the large size of the transgene, we successfully transduced around 20% of *ATM*-mutant cells. ATM expression and phosphorylation assays indicated that the neoprotein was functional in transduced cells, further reinforced by their restored capacity to phosphorylate direct ATM substrates such as p53 and their capability to repair radiation-induced DSBs. In addition, transduced cells also restored cellular radiosensitivity and cell cycle abnormalities. Our results demonstrate that lentiviral vectors can be used to rescue the intrinsic cellular defects of *ATM*-mutant cells, which represent, in spite of their limitations, a proof-of-concept for A-T gene therapy.

## Introduction

A-T (MIM# 208900) patients have cerebellar ataxia, heterogeneous immunodeficiency, frequent appearance of tumors, extreme radiosensitivity, endocrine abnormalities, incomplete sexual maturation, premature aging and vascular telangiectasias and impaired capacity to repair DSBs (1–4). This autosomal recessive disease is caused by mutations in the *ATM* gene (5), a key regulator in cell cycle and DNA repair mechanisms (1, 2). This gene, which contains 66 exons spanning over 150 kb of the genome with an open reading frame of 9,168 bp (6), encodes the 370 kDa ATM protein (5). Inactive ATM is found as dimers or tetramers that can be activated when recruited and anchored to DNA breaks by the sensor complex Mre11-Rad50-NBS1 (MRN) (7). Upon recruitment to DNA breaks by the MRN complex, ATM is autophosphorylated on Ser1981 (p-ATM), leading to its monomerization and subsequent activation of its kinase activity (8). Active ATM monomers phosphorylate downstream proteins that determine whether or not genomic instability can be prevented (9). Among them, the tumor-suppressor p53 protein is an important direct ATM substrate (10, 11), which partially explains cell cycle abnormalities observed in A-T cells (12). Upon activation, ATM phosphorylates histone H2AX on Ser139 (13), thereafter named γ-H2AX, which recruits additional DNA repair complexes at DSBs. Indeed, γ-H2AX accumulates in the vicinity of DNA breaks and can be readily detected by immunofluorescence forming characteristic foci in the nucleus (14). Hence, survival of damaged cells will depend upon the capability of these DNA repair mechanisms to properly correct DNA breaks.

Gene therapy is a valid strategy to treat patients suffering from several primary immunodeficiencies. Prior studies demonstrated that introduction of wild type *ATM* cDNA into ATM ^−^/^−^ human fibroblasts resulted in functional expression of the neoprotein, as revealed by restoration of ATM kinase activity and cell cycle abnormalities (15, 16). Likewise, intracerebellar injection of vectors into A-T animal models produced sustained protein expression in Purkinje cells (16, 17). Furthermore, transplantation of normal bone-marrow progenitors into *Atm*-mutant animals led to reconstitution of the hematopoietic system, thereby preventing the development of lymphomas (18). These studies introduce the concept that A-T patients could benefit from bone-marrow repopulation with corrected progenitors and integration of a WT *ATM* cDNA into cerebellar cells. The clinical application of these findings, however, is hampered by the delivery system required, as the length of the *ATM* cDNA (9.1 kb) prevents efficient packaging in commonly used vectors such as oncoretroviruses. This is why previous studies relied on the use of Herpes Simplex Virus Class 1 (HSV-1) amplicon vectors (15, 17, 18) or HSV/Adeno-associated (AAV) hybrid amplicon vectors (19), which can carry large cDNAs. These vectors, however, are non-integrative and the expression of the therapeutic protein is therefore transient; in addition, serious unresolved issues related to their pathogenicity and/or immunogenicity persist and the biosafety of these vectors is currently under intense scrutiny (20, 21). Furthermore, very recent data reveal severe adverse effects of AAV in non-human primates, which showed severe liver and sensory neuron toxicity (22).In addition, the biological significance of the aforementioned reconstitution studies are complicated by the limitations of the existing A-T animal model. Although the available *Atm*-deficient mice reproduce some of the defects found in A-T patients, it is unfortunate that they are not ataxic, and therefore, do not recapitulate the hallmark defect of the disease (23). Because of these limitations and biosafety concerns, gene therapy prospects for A-T patients remain somber.

Lentiviral vectors transduce resting cells without previous stimulation or addition of cytokines, can avoid silencing when controlled by adequate promoters and show little genotoxicity (24). Although lentiviral titers diminish as the size of the cDNA increases (25), some methods allow the production of lentiviral vectors containing large inserts (26). Third-generation lentiviral vectors consist, in addition to the therapeutic plasmid, in two separate packaging plasmids and another encoding the envelope protein (27). Although not completely risk-free, it is generally accepted that these vectors have improved biosafety because the probability of viral recombination is diminished (28), and therefore, are clinically relevant. In this study we demonstrate that, despite relatively modest viral titers, our lentiviral vector reconstitutes the ATM cellular phenotype, an initial step for the development of future preclinical studies for gene therapy in A-T patients.

## Methods

### Cells and culture media

GM07481 A-T fibroblasts, bearing an uncharacterized *ATM* mutation, were obtained from the Coriell Institute (Camden, NJ), whereas their healthy counterparts HFF-1 were from ATCC (SCRC-1041) and were both maintained in high-glucose DMEM media (Gibco, Paisley, United Kingdom), supplemented with 10% Fetal Bovine Serum (FBS) (Gibco), 1 mM L-glutamine and 100 μg/ml of penicillin-streptomycin. The amphotropic Phoenix-AMPHO cells and the human embryonic kidney (HEK) 293T cells were cultured as above.

### Lentiviral vector construction and production

**Figure 1:**
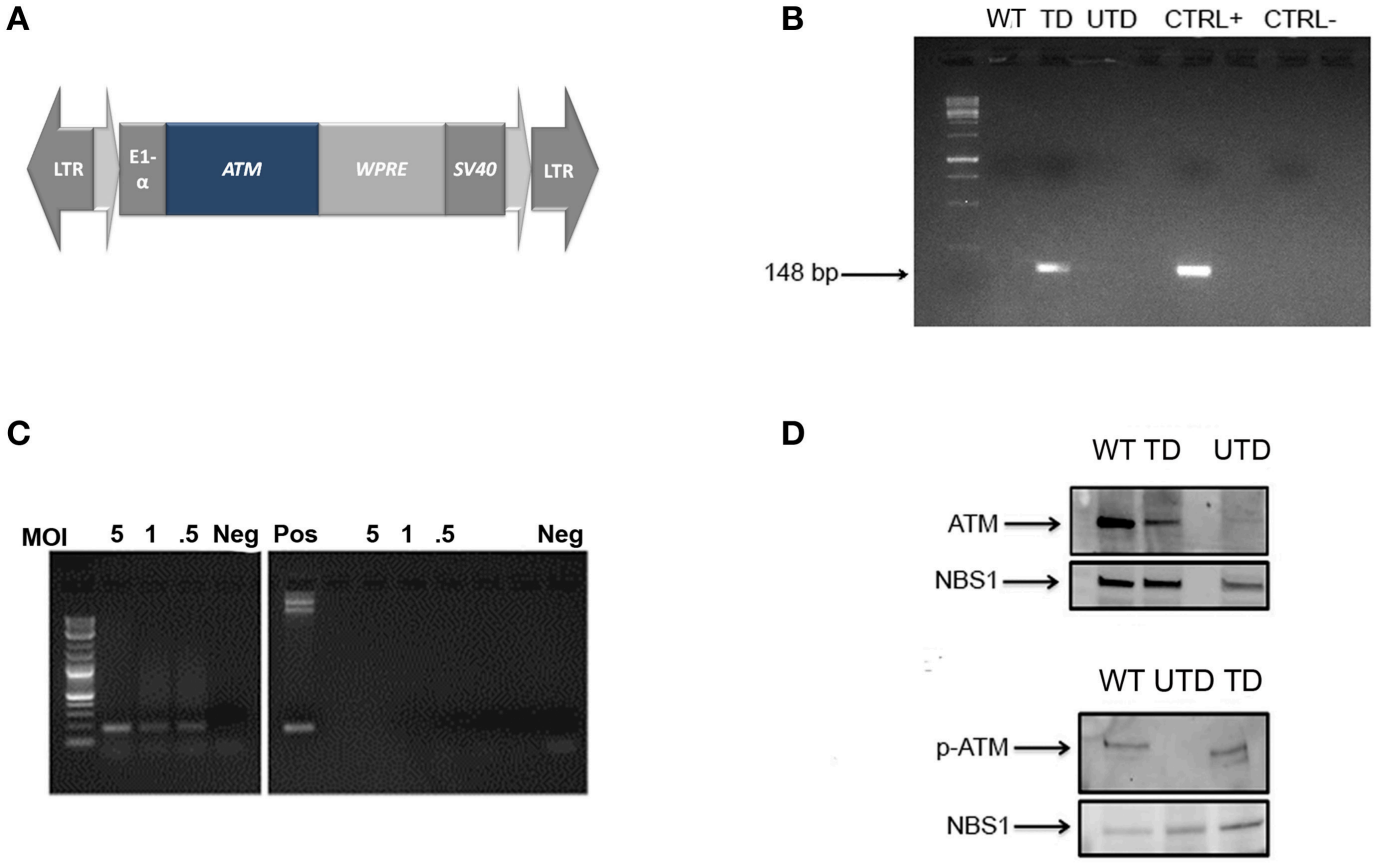
Construction and expression of a lentiviral vector containing a normal ATM cDNA. **(A)** Map of the lentiviral vector containing the full-length cDNA insert of the *ATM* gene WPRE: Woodchuck Hepatitis Virus Post-transcriptional Regulatory Element. **(B)** Integration of the therapeutic gene into the genomic DNA of healthy (WT) transduced (TD) and untransduced (UTD) cells determined by PCR using vector-specific sequences. ThATM plasmid DNA was used as positive control. **(C)*** ATM* mRNA expression in transduced cells at indicated MOI (5, 1, and 0.5). Negative controls (right-hand gel) used equivalent amounts of non-retrotranscribed templates and positive control used the pThATM plasmid as amplification template. Neg: no template. **(D)** ATM expression in nuclear extracts of native ATM (top) or Ser1981 p-ATM (bottom) after cell irradiation. The nuclear protein NBS1 was used as loading control. WT, HIFs derived from healthy subjects; UTD, untransduced A-T HIFs; TD, A-T HIFs cells transduced with the ThATM lentiviral vector.

To construct the ATM lentiviral vector, the full-length *ATM* cDNA contained in a pcDNA3.1 plasmid (Addgene, #31985, Cambridge, MA) was excised and inserted by a three-step subcloning strategy into the lentiviral plasmid pThOKSIM to generate the pThATM plasmid (Figure 1A) under the control of the human elongation factor 1 alpha (EF1α) promoter. Viral particles were produced by co-transfecting HEK 293T cells with plasmids pThATM, pHDM.G, pHDM-Tat, pRC/Rev, and pHDM-Hgm2, encoding for the ATM; Vesicular Stomatitis Viral G-protein (VSV-G); trans-activator of transcription (tat); regulator of expression of virion proteins (rev); and group antigen/polymerase (gag/pol) products, respectively (19.2; 1.92; 0.96; 0.96, and 0.96 μg of plasmid DNA) using lipofectamine-2000 (Invitrogen). Viral particles were collected, concentrated by ultracentrifugation (29, 30) and used immediately.

### Generation of normal and ATM-deficient human immortalized fibroblasts (HIFs)

The catalytic subunit of human telomerase on vector pBABE-hygro-hTERT plasmid (Addgene, #1773) was packaged into retroviral particles at 32°C using the amphotropic Phoenix A cell line and Fugene-6 (Promega, Madison, WI). Wild Type and ATM-mutant HIFs were transduced and resistant colonies pooled and expanded after 15 days of hygromycin selection (Sigma Chemical, Saint Louis, MO).

### Transduction of cells

ATM-deficient HIFs were seeded in 24-well plates (7.5 × 10^4^ cells/well) and 24 h later viral supernatants were added in the presence of 5 μg/ml of polybrene (Sigma).

### Antibodies

The following monoclonal antibodies (mAbs) were used: anti-ATM 1A1 (Santa Cruz Biotechnology, Santa Cruz, CA); anti-ATM pSer1981 mAb (10H11E12, Rockland, Givertsville, PA); anti-NBS1 (34/NBS1, BD Biosciences, San Jose, CA); anti-p53 (DO-1; Sigma); rabbit antiserum against phosphorylated p53 on Ser15 (p-p53) was obtained from Santa Cruz Biotechnology and rabbit polyclonal anti-γ-H2AX from Abcam (Cambridge, U.K.). The Hoechst 33342 reagent and the AlexaFluor 488 goat anti-mouse or goat anti-rabbit secondary antibodies were obtained from LifeTechnologies. Goat and mouse normal sera used in blocking steps were from Sigma.

### Lentiviral vector titration

Lentiviral vector titration was determined on genomic DNA (gDNA) by quantitative PCR (qPCR) as described (29, 31). Briefly, 293T cells were transduced as above with 1, 10, or 100 μl of viral supernatants, and after media replacement, were allowed to grow for 3 weeks prior gDNA extraction and analysis. Results were expressed as vector genomes/ml.

### PCR analysis of gDNA and calculation of number of integrations per cell

gDNA was isolated using the QIAamp DNA mini kit (Qiagen, Valencia, CA) 3 weeks after transduction of cells to avoid interference with non-integrated vectors. Confirmation of plasmid integration was determined by end-point PCR whereas the number of vector integrations per cell was determined by qPCR. gDNA (0.1 μg) was amplified using primers spanning vector-specific and *ATM* sequences: Forward: 5′-GACGATGACGACAAGATGCA-3′ and Reverse: 5′-TTTGGAATCTGAATGCCGAT-3′. End-point PCR conditions were: [5 m 95°C; 35x (45 s 95°C; 1 m 57°C; 1.5 m 72°C); 13 m 72°C]. The 116-bp PCR product was resolved in a 2% agarose gel, purified and sequenced. For qPCR, 0.2 μg of gDNA was amplified in a 7500 Real-Time PCR System (Applied Biosystem, Foster, CA) using the Fast Start Universal SYBR Green Master (Roche). qPCR was performed on transduced cells using the vector-specific primers indicated above, whereas gDNA from wild type cells was amplified with a pair of validated primers spanning *ATM* intron 23 and exon 24 as follows: F-ATM-I23: 5′-TGTTCCAGGACACGAAGGGAGA-3′ and R-ATM-E24: 5′- CAGGGTTCTCAGCACTATGGGA-3. qPCR conditions were [2 m 95°C; 40x (20 s 95°C; 30 s 60°C; 30 s 72°C)]. Human β-actin was used to determine the human allele copy number in each allele and amplified using primers: F-ACT: 5′-AGCCTCGCCTTTGCCGATCC-3′; and R-ACT: 5′-ACATGCCGGAGCCGTTGTCG-3′. The number of vector integrations was calculated by interpolation from a standard curve constructed using 10-fold increases (from 10 to 10^7^ copies) of plasmid ThATM.

### Analysis of RNA

Total RNA was extracted from transduced and ATM-deficient cells using TRIzol (Invitrogen), reverse transcribed with the High-Capacity cDNA reverse transcription kit (Applied Biosystems) and analyzed by end-point and quantitative PCR using the primers indicated above. Conditions for end-point PCR were [4 m 94°C; 35x (20 s 94°C; 20 s 52°C; 45 s 72°C); 10 m 72°C] and for qPCR [10 m 95°C; 40x (30 s 95°C; 60 s 55°C; 60 s 72°C)].

### Western blotting analyses

Nuclear lysates from cells non-irradiated and irradiated with a ^137^Cs MARKI irradiator (JL Shepherd, San Fernando, CA) were extracted using the NER-PER^TM^ system (Life Technologies) following manufacturer's instructions. Solubilized extracts (50 μg per lane) were loaded onto 4%-15% Mini Protean TGX Precast gels (BioRad), electrophoresed and electrotransferred onto a PVDF membrane (Trans-Blot Turbo Transfer system, BioRad) following manufacturer's recommendations for high m.w. proteins. Membranes were blocked with 10% non-fat milk in 0.1% PBS/Tween 20 or TTBS (50 mM TRIS, 150 mM NaCl, pH 7.6) for 1 h and incubated overnight with pre-determined optimal concentrations of primary antibodies; after washes, membranes were incubated with secondary antibodies for 1 h at room temperature (RT), developed by chemiluminescence (SuperSignal West *Femto*, Thermo Scientific), recorded with a digital imaging system (Fujifilm Image Analyzer LAS-4000, Tokyo, Japan) and analyzed with the MultiGauge software.

### Confocal microscopy

Cells were pre-seeded in 8-well chamber slides (30 × 10^3^ per well), irradiated and cultured for indicated times. For γ-H2AX staining, cells were starved for 12 h prior irradiation. Cells were fixed at end-points with either 3% paraformaldehyde and 2% sucrose for 10 m or 100% methanol for 10 m (for γ-H2AX immunostaining), permeabilized, blocked and incubated with primary antibodies in PBS supplemented with 2% Bovine Serum Albumin and 10% FBS for 1 h at 37°C (for p-ATM and p-p53 staining) or overnight at 4°C in a solution of PBS; 2% BSA; 10% normal goat serum and 0.3 M glycine (for γH2AX staining). After washes, cells were incubated with Alexa Fluor 488-labeled secondary antibodies for 1 h at RT. Cell nuclei were counterstained with Hoeschst 33342 (Thermo Fisher). Confocal microscopy used a Zeiss LSM 710 Confocal Laser Scanning Microscope. At least 200 nuclei from each sample were counted and those containing ≥4 foci were considered positive. Results are reported as number of foci per cell (average of total foci counted/average of total nuclei); percentages of cells with foci (for p-ATM and p-p53) or percentage of repairing cells (for γ-H2AX) (% Repairing cells = ∑Total counted nuclei/∑ nuclei positive).

### Colony survival assays

*In vitro* radiosensitivity assays were performed as described (32) using increasing numbers of cells (from 500 to 10,000 for normal and transduced cells; 4,500 to 30,000 for untransduced). Cells were irradiated and cultured for 2 weeks, fixed in PBS/0.5% formaldehyde/5% glutaraldehyde, stained with crystal violet and photographed. Clusters containing over 50 cells were considered colonies (123 pixels) as analyzed with ImageJ software. The total area occupied by colonies produced by irradiated cells was quantified and compared to that from untreated cells in each group. Statistical analysis and survival fractions (SF) were calculated as described (32). According to established values (33), cells were considered radiosensitive (SF <21%); intermediate radiosensitivity (SF 21-35%) or normal (SF≥36%).

### Cell cycle analyses

Cells were irradiated with 4 Gy, cultured for 18 h and cell cycle profiles were determined by flow cytometry after Propidium Iodide staining as described elsewhere (34) using a BD FACSCanto II flow cytometer (Becton Dickinson, San Jose, CA).

## Results

### Transduced human-immortalized fibroblasts (HIFs) from A-T patients express a functional ATM protein

To test whether or not A-T patients could benefit from integrative gene therapy, we constructed a third-generation lentiviral plasmid containing the *ATM* cDNA (pThATM; Figure 1A). Vector packaging typically yielded 5 × 10^6^ vector genomes/ml after viral concentration as indicated. HIFs from A-T patients or healthy individuals (WT) (35) were established, transduced with increasing MOIs (Figure 1B) and vector integration efficiency determined using pThATM-specific primers. Amplification of exogenous ATM was detected only in transduced cells, whereas amplification products were not observed in neither untransduced nor WT cells (Figure 1B). Cells transduced at MOI = 5 integrated 0.4 copies of exogenous *ATM* per cell, and the resulting cell line was used, for consistency, throughout the study. Notably, integrated vectors efficiently transcribed the ATM transgene in a dose-dependent manner, as detected by RT-PCR (Figure 1C). Nuclear extracts from HIFs cells were analyzed by Western Blotting, and expression of the ATM protein was detected in mutant cells after lentiviral transduction, although ATM expression was weaker than that of WT control cells (Figure 1D, top panel). The lentiviral-expressed ATM protein was functionally active, as demonstrated by the appearance of p-ATM upon irradiation of transduced cells (Figure 1D, bottom panel).

**Figure 2:**
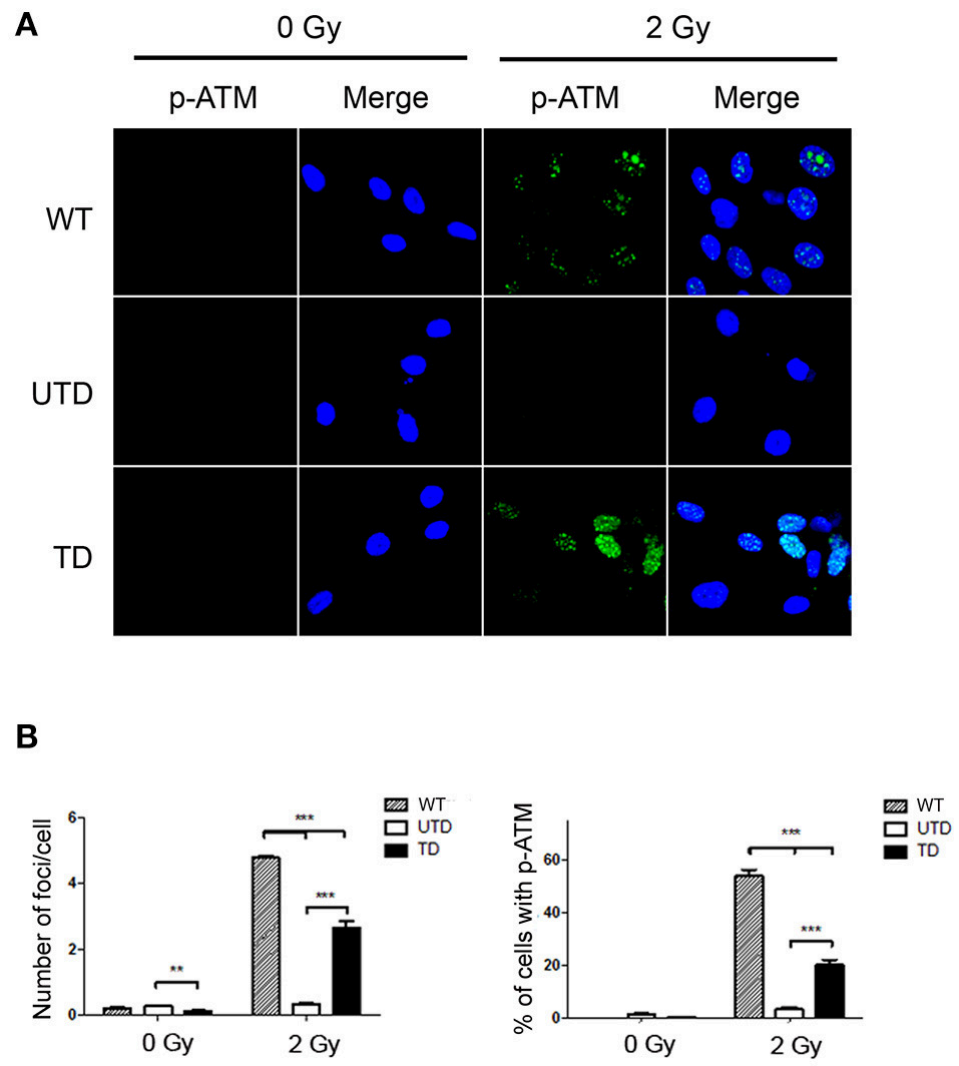
Accumulation of p-ATM foci in A-T HIFs transduced with the lentiviral vector ThATM. **(A)** Confocal microscopy analyses of wild type (top images), untrasduced ATM-deficient HIFs (UTD, central images) and transduced ATM-deficient HIFs (TD, bottom pictures) stained with the anti p-Ser1981ATM mAb (p-ATM, green) either irradiated with 2 Gy (right) or without irradiation (left). The nuclei of cells were counterstained with DAPI (blue). Random pictures (p-ATM and a merged image with the DAPI staining) of fields from a representative experiment out of three are shown. **(B)** Quantification of the number of p-ATM foci per cell (left) and the percentage of cells containing p-ATM foci (right). Blind quantifications of at least 200 cells per each of the three experiments were analyzed. Bars indicate mean ± standard deviation of values. **p < 0.01; ***p < 0.001 using the two-tailed *t*-test for paired observations. WT, HIFs derived from healthy subjects; UTD, untransduced A-T HIFs; TD, A-T HIFs cells transduced with the ThATM lentiviral vector.

To estimate the percentage of reconstituted cells upon transduction, we used confocal microscopy to detect p-ATM foci before and after ionizing irradiation. Irradiated untransduced cells had no detectable p-ATM foci, whereas, at stark contrast, these were readily detected in transduced cells (Figure 2A). Blind quantification of the number of cells containing p-ATM foci (Figure 2B, left-hand graph), as well as, the number of foci per cell (Figure 2B, left-hand graph) indicated that p-ATM foci were found in 19.3 ± 5.1% of transduced cells and in 50.8 ± 5.1% of WT cells treated in parallel as controls (Figure 2B, left hand graph). The average number of ATM foci in reconstituted cells was, however, lower than that found in WT cells (Figure 2B, left-hand graph).

### A-T deficient cells transduced with ThaTm lentiviruses can repair irradiation-induced DSBs

**Figure 3:**
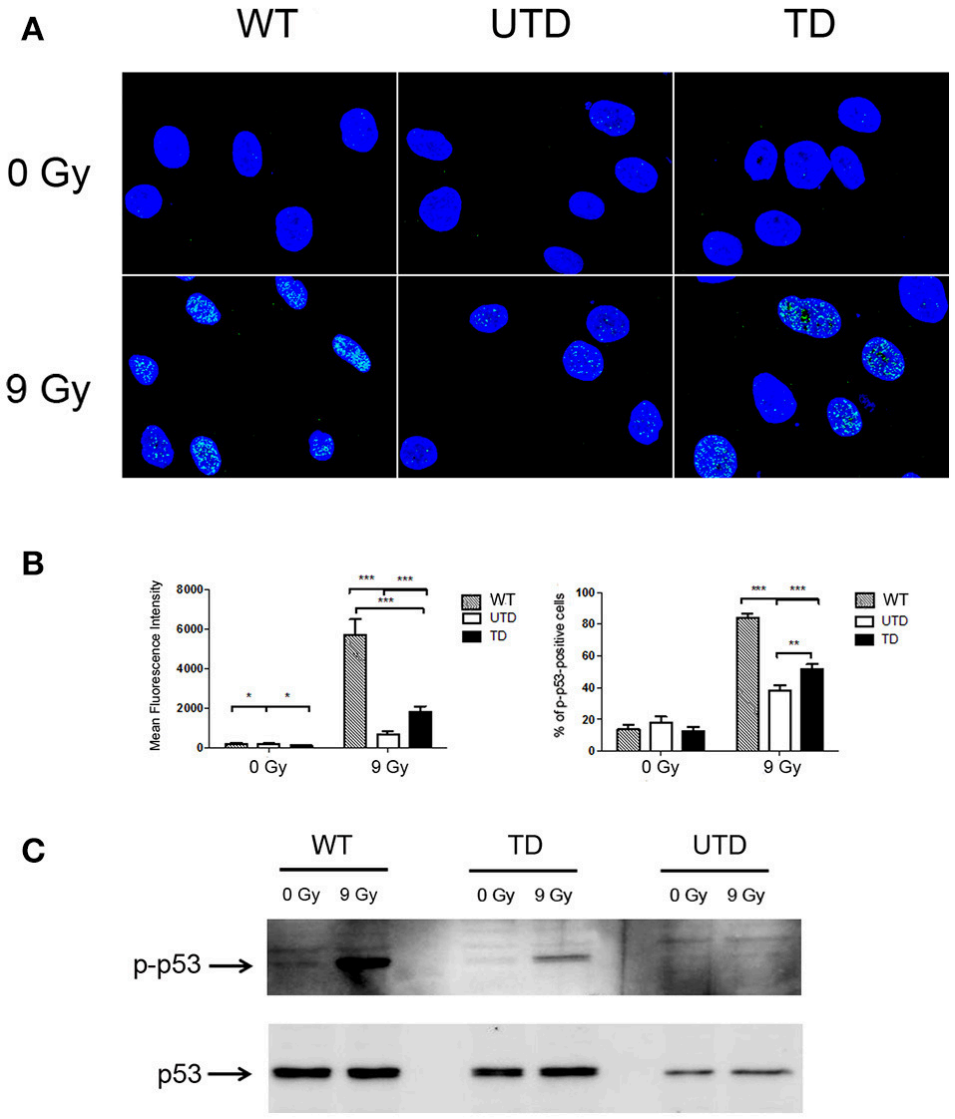
Foci formation of p-p53 in WT and A-T HIFs transduced with the ThATM lentiviral vector. **(A)** Confocal microscopy analysis of wild type (top figures), untransduced A-T HIFs (UTD, central figures) and transduced A-T HIFs (bottom figures) stained with an anti-p-p53 mAb (green) after irradiation of cells with 9 Gy. The nuclei of cells were counterstained with DAPI (blue) and merged images are shown. Random fields from a representative experiment out of three are shown. **(B)** Graphs showing quantification of the Mean Fluorescence Intensity and the percentage of cells with p-p53 foci. Blind quantifications of at least 200 cells per each of the three experiments were conducted to determine the Mean Fluorescence Intensity (left-hand graph) and the number of cells containing p-p53 foci (right-hand graph). Bars indicate mean ± standard deviation of values. *p < 0.05; **p < 0.01; ***p < 0.001 using the two-tailed *t*-test for paired observations. **(C)** Western Blot analysis of p-p53 expression. Extracts from untreated or irradiated cells were analyzed by Western Blot and probed with antibodies against p-p53 (top gel) or native p53 (used as a loading control; bottom gel). WT, HIFs derived from healthy subjects; UTD, untransduced A-T HIFs; TD, A-T HIFs cells transduced with the ThATM lentiviral vector.

The tumor suppressor gene p53 is a direct downstream substrate of ATM (10). Because of this, and to accumulate evidence about the functionality of the ThATM vector, we tested whether ATM-mediated p53 phosphorylation could be detected in transduced cells upon γ-irradiation. As expected, intranuclear foci of p-p53 were observed in irradiated WT cells, indicating accumulation of activated p53 on chromatin (Figures 3A,B, left-hand top panels and graph). However, and because p53 is not exclusively phosphorylated by ATM, a residual activation of p53 was also distinguished in irradiated ATM-deficient cells (Figures 3A,B; top central panels and right-hand bottom graph). The fluorescence intensity detected in untransduced cells was much lower than that observed in WT HIFs, even though p-p53 foci were detected in about 40% of cells. We observed that transduced cells had a higher percentage of p-p53 foci, which was consistent with the estimated percentage of cells efficiently transduced; additionally, a clear increase in the fluorescence intensity of p-p53 foci was observed (Figure 3B, left-hand graph). These results were confirmed by western-blotting, revealing the appearance of a clear p-p53 band in transduced cells upon irradiation (Figure 3B). Expression levels of p-p53 correlated with the transduction efficiency of HIFs, although p-p53 expression values were lower than those of WT cells (Figure 3C). As expected, only traces of p-p53 could be detected in untransduced cells.

**Figure 4:**
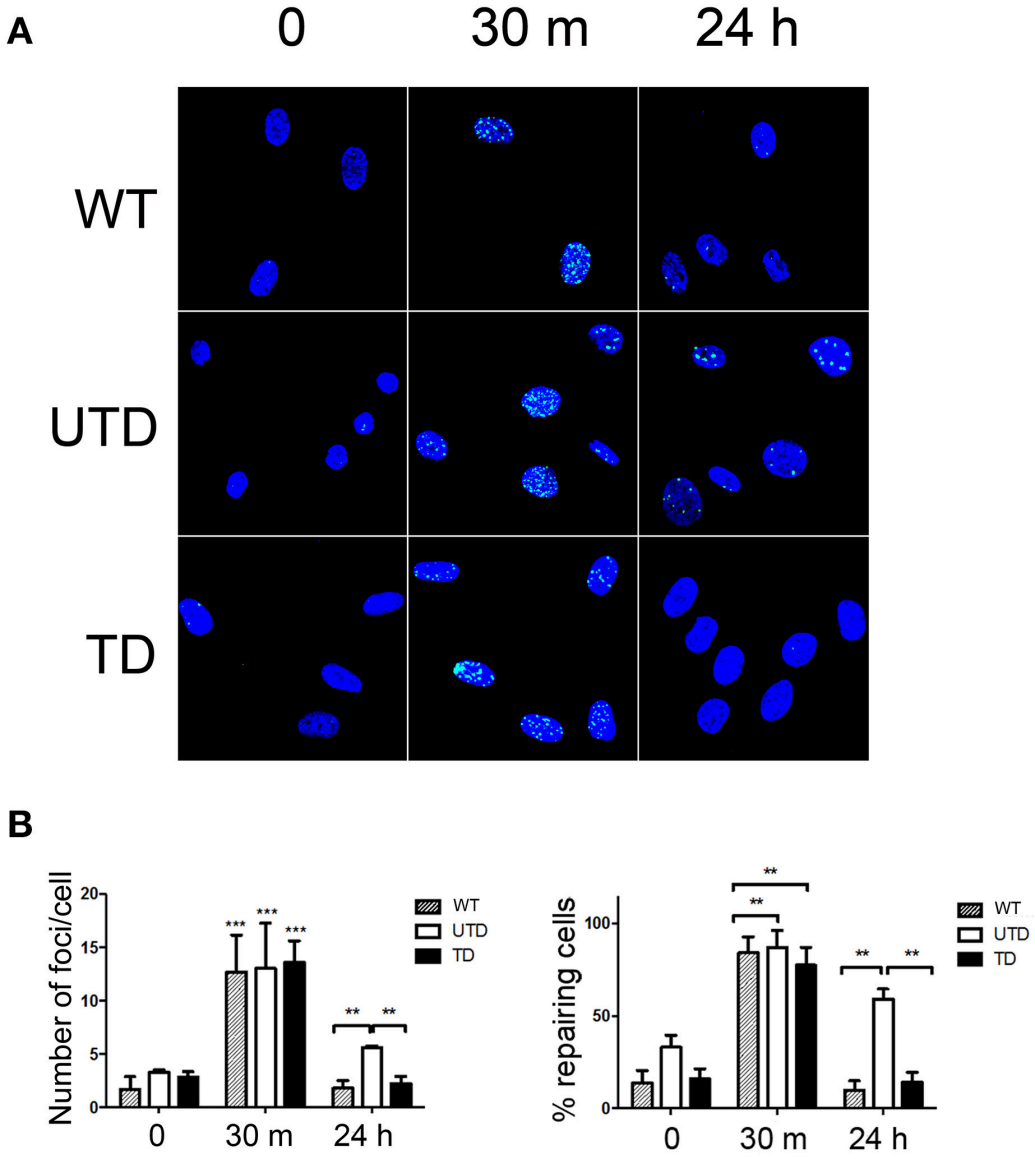
A-T HIFs cells transduced with the ThATM lentiviral vector recover the ability to repair irradiation-induced DSB as determined by γ-H2AX foci formation. **(A)** WT HIFs (top pictures), untransduced A-T HIFs (middle pictures) and transduced A-T HIFs (bottom pictures) with the lentiviral vector, were irradiated with 2 Gy and γ-H2AX foci formation (green) analyzed by confocal microscopy at indicated timepoints. Random fields from a representative experiment out of three are shown. **(B)** Quantification of the percentage of cells with γ-H2AX foci as an indication of ongoing DNA repair activity (right graph) and of the number of γ-H2AX foci per cell (left graph). Blind quantifications of at least 200 cells per each of the three experiments were carried out. The bars indicate mean ± standard deviation of values. **p < 0.01; ***p < 0.001 using the two-tailed *t*-test for paired observations. WT, HIFs derived from healthy subjects; UTD, untransduced A-T HIFs; TD, A-T HIFs cells transduced with the ThATM lentiviral vector.

Irradiation-induced DSBs result in phosphorylation of the specialized histone H2AX on Ser139 [i.e., γ-H2AX (36)]. Phosphorylated γ-H2AX induces the formation of protein conglomerates of damage sensors in the proximity of the lesions that can be detected as intranuclear γ-H2AX foci, whose formation is a critical step to efficiently trigger DNA repairing mechanisms. These γ-H2AX foci were detected in all cells 30 m after irradiation (Figure 4A, central column), and no differences were observed in the number of γ-H2AX foci per cell among WT (12.3 ± 3.8), untransduced (12.7 ± 3.9), or transduced (12.9 ± 3.7) HIFs (Figure 4B, left-hand graph). γ-H2AX is gradually dephosphorylated upon repairing of the induced DSBs. Whereas, γ-H2AX foci persisted 24 h after irradiation of untransduced A-T HIFs, these foci were no longer detected 24 h after irradiating WT or transduced HIFs (Figure 4A, right-hand top and bottom panels; Figure 4B, right-hand graph). Indeed, the persistence of γ-H2AX foci in 58.47 ± 4.96 untransduced cells strongly suggests that DSBs remain unrepaired (Figure 4A, right-hand central panel and Figure 4B, right graph).

### ATM deficient cells transduced with ThATM lentiviruses restore the radiosensitivity defect

**Figure 5:**
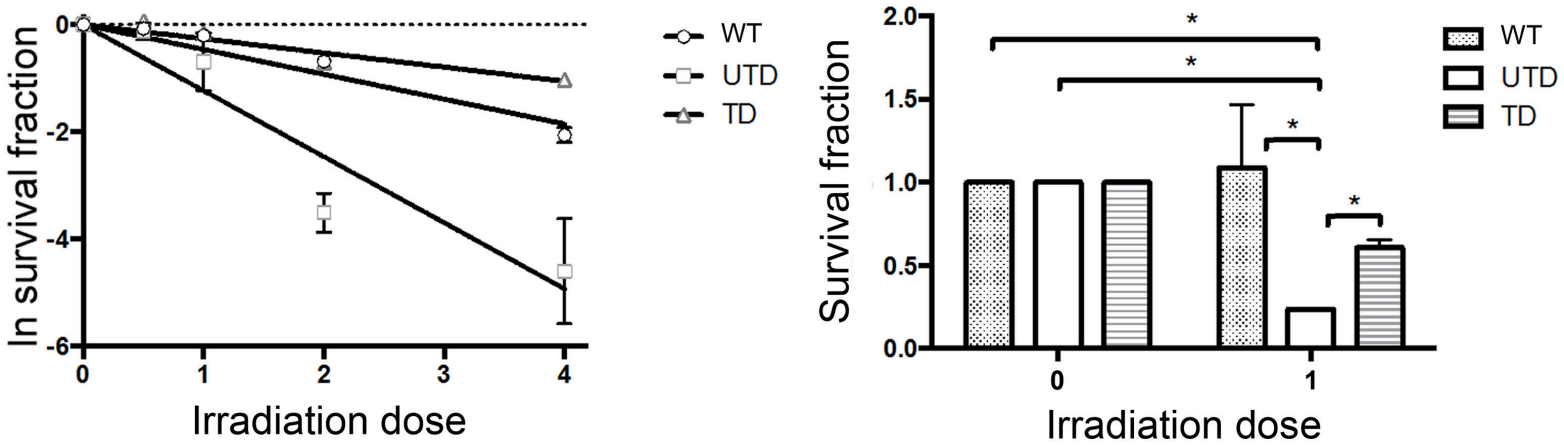
Colony Survival Assays showing restoration of radiosensitivity in A-T HIFs transduced with the lentiviral ThATM vector. WT and A-T HIFs (untransduced and transduced) were irradiated with each indicated doses and Colony Survival Assays were carried out in triplicates. Colony numbers were calculated using ImageJ software. The left graph represents the Naeperian Logarithm of the survival fraction adjusted to a linear model; and the right graph represents the survival fraction upon 1 Gy irradiation. Results were normalized to the data obtained with non-irradiated cells for each group, and assigned a value of 1. *p < 0.05 using the two-tailed *t*-test for paired observations. All other group comparisons showed non-significant differences. WT, HIFs derived from healthy subjects; UTD, untransduced A-T HIFs; TD, A-T HIFs cells transduced with the ThATM lentiviral vector.

Because radiosensitivity is a hallmark of in both A-T patients and cultured A-T cells (37), we performed colony-survival assays to determine whether ThATM lentiviral transduction rescue ATM-deficient cells from this defect. As expected, *Napierian logarithm* value analyses of the survival fraction of irradiated cells revealed a strong radiosensitivity of A-T HIFs (UTD, Figure 5, left graph). Transduction of A-T-derived cells with the ThATM lentiviral vector revealed a significant increase in the fraction of surviving cells (TD, Figure 5). Transduced HIFs reversed their inherent radiosensitivity to levels approaching those observed in WT cells (Figure 5).

### Cell cycle abnormalities are restored in cells transduced with ThATM lentiviruses

**Figure 6:**
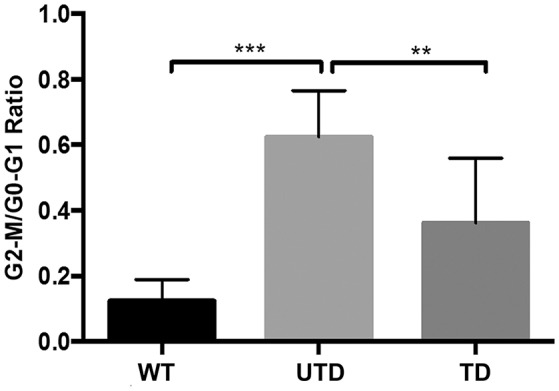
Reconstitution of cell cycle abnormalities in A-T HIFs transduced with the ThATM lentiviral vector. WT and A-T HIFs (untransduced and transduced) were irradiated with 4 Gy and further cultured for 18 h prior to analyse their cell cycle phases distribution by flow cytometry (using propidium iodide staining, see Methods). The percentage of cells in each phase of the cell cycle was determined and the graph shows the mean ± S.D of the G2-M/G0-G1 ratio of three independent experiments. **p < 0.01; ***p < 0.001 using the two-tailed *t*-test for paired observations. All other group comparisons showed non-significant differences. WT, HIFs derived from healthy subjects; UTD, untransduced A-T HIFs; TD, A-T HIFs cells transduced with the ThATM lentiviral vector.

Because ATM is a key regulator of cell cycle checkpoints, particularly those operating at G1/S and G2/M interphase, cells from A-T patients are normally arrested at G2/M phase 24 h after irradiation (38). We therefore studied whether or not these defects could also be restored in lentiviral-transduced A-T HIFs cells. The quantification of accumulated cells at G2/M phase is estimated by the ratio of G2-M/G0-G1, previously used to demonstrate ATM functional reconstitution of defective cells (15). As expected, untransduced A-T HIFs cells were arrested at the G2/M phase 18 h post-irradiation, as revealed by a G2-M/G0-G1 ratio of 0.62 ± 0.08 (Figure 6). In sharp contrast, WT cells progressed through cell cycle and had a much lower ratio (G2-M/G0-G1 = 0.12 ± 0.03). Transduced cells, importantly, showed an intermediate G2-M/G0-G1 ratio (G2-M/G0-G1 = 0.36 ± 0.11), consistent with the estimated percentage of transduction efficiency achieved with ThATM lentiviruses (Figure 6).

## Discussion

A-T patients sustain a high morbidity and mortality because of the severity of the disease and the lack of effective treatments. Although several organs are affected, abnormalities in hematopoietic cells play prominent roles in the general context of the disease (1). Reconstitution of hematopoietic bone marrow progenitors in a murine model of the disease resulted in extended lifespan of transplanted mice (18), and two recent reports described significant clinical benefits in two A-T patients who received a bone marrow transplant to treat concomitant Acute Lymphoblastic Leukemia (39) and non-Hodgkin lymphoma (40).

Recent improvements on gene delivery vectors are the basis to evaluate the efficacy of gene therapy protocols in the treatment of complex diseases such as A-T. Indeed, the delivery of the *ATM* cDNA into mutant mice was successful (15, 19) and amelioration of neurological symptoms after intracranial vector injection was observed (17). The vectors used, HSV-1 and HSV/AAV amplicons, were efficient in packaging the large *ATM* cDNA but, unfortunately, their characteristics (summarized in the introduction) make them unlikely tools to treat A-T patients due to their challenged biosafety (22) and non-integration into the host genome.

We constructed a third-generation lentiviral vector (27) containing the *ATM* cDNA to test whether or not this vector was able to rescue A-T cell functions *in vitro*. HIFs were chosen as targets because they represent a stable experimental system that mimic the intrinsic defects of the disease observed in primary A-T cells (35). Vector integration into mutant cells resulted in expression of a functional ATM protein, as revealed by its phosphorylation upon irradiation, a key indicator of a correct activation. Moreover, confocal microscopy detected p-ATM foci of normal shape in more than 20% of cells, which is consistent with the lentiviral titers and vector copy numbers achieved and the expected transduction efficiency.

The analysis of γ-H2AX foci is considered a new cornerstone assay to evaluate ATM function because its sensitivity and reproducibility (41, 42). The restoration of this function in transduced cells and their capacity to phosphorylate the ATM-direct substrate p53 are further evidence that transduced cells were repaired by the therapeutic vector generated in this study. Upon recruitment of γ-H2AX at DSBs the cascade of DNA repair processes is triggered. Whereas 24 h after irradiation γ-H2AX foci could be detected in ATM-deficient cells, suggesting the presence of abundant unrepaired DSBs, these foci were not detectable in ATM-transduced cells. This is an indication that complementation with the vector generated in this study resets cells to a pre-damage homeostatic dephosphorylated situation (43). The ability of transduced cells to repair DSBs was reinforced by the reversal of the A-T radiosensitivity and cell cycle abnormalities. The levels of rescue and complementation of A-T defects observed with ThATM are similar to that previously observed in studies that treated ATM-deficient cells with small molecule compounds that allow read-through over Premature Termination Codons (PTCs) (42, 44). Unfortunately, the percentage of patients suffering from A-T due to the presence of PTCs in the *ATM* gene that could benefit from this chemical treatment is very low in some cohorts (45), and hence, gene therapy should be considered as an alternative and perhaps better therapeutic option.

Our results indicate that although lentiviral titers are low because of the size of the *ATM* cDNA, gene therapy reconstitutes cellular defects of A-T cells. A recent study showed that lentiviral vectors can also rescue defective Duchenne Muscular Distrophy cells, but because the size of the dystrophin gene is even higher than that of *ATM* (15 kb), viral titers were concurrently lower than those obtained in this work (46). Gene therapy for A-T showed promise after correcting hematopoietic progenitors in animal models of the disease, further reinforced by the outcome of bone marrow transplants performed in A-T patients due to concomitant hematopoietic malignancies (39, 40). Although the disease is not restricted to the hematopoietic system, this treatment led, surprisingly, to an improvement of the neurological defects. This unexplained observation suggests pleiotropic clinical benefits derived from reconstituting the hematopoietic system, which should be interpreted within the newly proposed ATM functions extending beyond DNA repair (47–49). Because of this systemic effect, it is possible to speculate that gene therapy could ameliorate neurological functions in A-T patients. In addition, and as demonstrated in animal models (50), lentiviral vectors could, in theory, be delivered into the brain of A-T patients to reach Purkinje cells. Human clinical trials, however, require sharp improvements in viral packaging efficiency. Fortunately, continuous efforts are currently underway in this area, including the development of stable packaging cell lines (51) or codon-optimized vectors. On the other hand, it is possible that low-levels protein expression will nevertheless result in clinical benefit for the patients. In support of this idea is the fact that leaky functional expression (as little as 9% of the protein levels found in normal cells) of the mutated gene causing certain primary immunodeficiencies such as the Wiskott-Aldrich syndrome, resulted in a mild presentation of the disease (52). This is also the case of A-T, where an elegant study of a large cohort of patients demonstrated that those individuals retaining residual ATM kinase activity presented mild clinical symptoms, with greatly diminished neurological affectation and prolonged survival (53).

In summary, we provide evidence that a third-generation lentiviral vector containing the *ATM* cDNA is able to reconstitute disease-associated defects in transduced patient cells. In spite of the efficiency limitations due to the large size of the transgene, our results are proof-of concept for integrative gene therapy in A-T, an approach that requires further studies to pave its way for future trials in humans. Although fibroblasts are relatively permissive cells, the transduction efficiency achieved in hematopoietic precursors is moderately lower. Nevertheless, improving the delivery efficiency in hematopoietic precursors, as well as obtaining solid *in vivo* data in an animal model that does not faithfully mimics the human disease are significant hurdles and key areas for immediate work.

## Ethics statement

Human cells were obtained from public repositories and handled according to the recommendations of the University of Granada's Ethics Committee on Human Experimentation. Primary cells or tissues were not used in this study, and therefore a consent to participate is not applicable.

## Author contributions

DC, ST-R, GC-P, EB-J, and MM-L performed experiments. JLG-P and IJM directed research and wrote the paper.

### Conflict of interest statement

The authors declare that the research was conducted in the absence of any commercial or financial relationships that could be construed as a potential conflict of interest.
